# Sucrose in Cyanobacteria: From a Salt-Response Molecule to Play a Key Role in Nitrogen Fixation

**DOI:** 10.3390/life5010102

**Published:** 2015-01-06

**Authors:** María A. Kolman, Carolina N. Nishi, Macarena Perez-Cenci, Graciela L. Salerno

**Affiliations:** Instituto de Investigaciones en Biodiversidad y Biotecnología (INBIOTEC-CONICET) and Fundación para Investigaciones Biológicas Aplicadas (FIBA), Mar del Plata B7600DHN, Argentina; E-Mails: mkolman@fiba.org.ar (M.A.K.); cnishi@fiba.org.ar (C.N.N.); mperezcenci@fiba.org.ar (M.P.-C.)

**Keywords:** sucrose metabolism, compatible solutes, salt tolerance, glycogen, nitrogen fixation, signal molecule

## Abstract

In the biosphere, sucrose is mainly synthesized in oxygenic photosynthetic organisms, such as cyanobacteria, green algae and land plants, as part of the carbon dioxide assimilation pathway. Even though its central position in the functional biology of plants is well documented, much less is known about the role of sucrose in cyanobacteria. In those prokaryotes, sucrose accumulation has been associated with salt acclimation, and considered as a compatible solute in low-salt tolerant strains. In the last years, functional characterizations of sucrose metabolizing enzymes, metabolic control analysis, cellular localization of gene expressions, and reverse genetic experiments have revealed that sucrose metabolism is crucial in the diazotrophic growth of heterocystic strains, and besides, that it can be connected to glycogen synthesis. This article briefly summarizes the current state of knowledge of sucrose physiological functions in modern cyanobacteria and how they might have evolved taking into account the phylogenetic analyses of sucrose enzymes.

## 1. Introduction

Cyanobacteria are among the most diverse groups of prokaryotic organisms that perform oxygenic photosynthesis. In a long evolutionary history, cyanobacterial diversification was one of the most important increases in physiological and morphological complexity of the prokaryotes [1]. As the result of their remarkable capacity to adapt to environmental changes by the acquisition of elaborate growth strategies [2], modern cyanobacteria exhibit a wide range of morphologies (*i.e.*, unicellular to various multicellular organizations) and can be found occupying marine or freshwater aquatic environments or terrestrial ecosystems [2]. Particularly, the majority of free-living forms are abundant in waters with rapid and significant salinity fluctuations and water status [3]. To cope with these variations, cyanobacteria have developed salt acclimation mechanisms that involve the active extrusion of toxic ions and the accumulation of compatible solutes, such as sucrose, trehalose, glucosylglycerol, glucosylglycerate and glycine betaine [4]. The first recognized physiological function for sucrose was its salt stress-induced accumulation, which was well documented in the early 80s [5,6,7]. An extensive screening of cyanobacteria revealed that freshwater strains with rather low salt tolerance mainly accumulate sucrose. An additional role was proposed for sucrose as a carbon carrier molecule from the vegetative cell to the heterocyst in filamentous nitrogen-fixing strains [8]. However, a better understanding of its functions was gained after the functional identification of sucrose metabolism-related genes in unicellular and heterocyst-forming strains [9,10,11,12].

This review summarizes the current state of knowledge of sucrose roles in cyanobacteria, as a stress-response molecule, its relationship with glycogen metabolism, and most outstandingly, as a crucial molecule in filamentous heterocyst-forming strains.

## 2. Sucrose Metabolism in Cyanobacteria

### 2.1. Sucrose Enzymes

In contrast to plant enzymes [13,14], the proteins involved in sucrose metabolism in cyanobacteria, more recently described, have been studied to a lesser extent.

**Figure 1:**
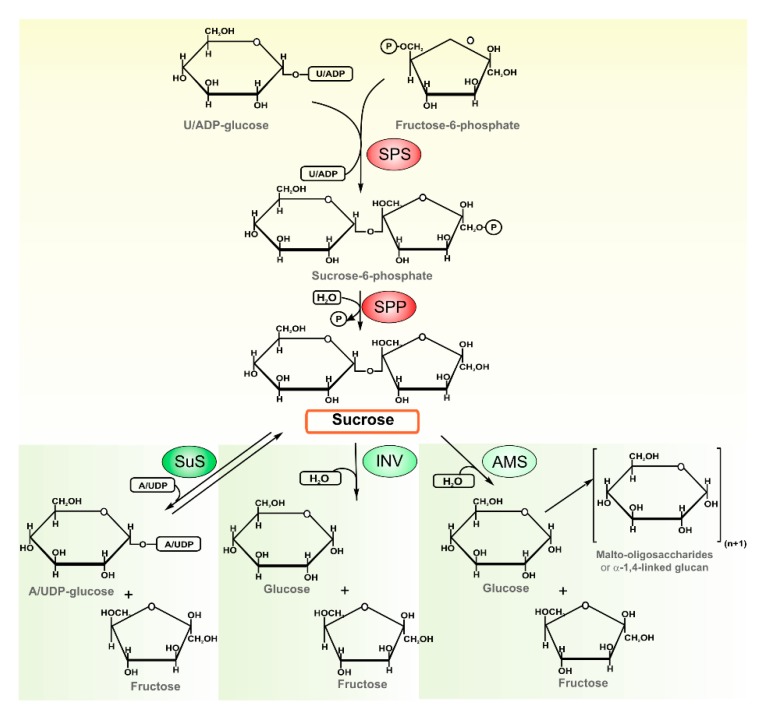
Schematic representation of sucrose metabolism in cyanobacteria. Sucrose biosynthesis involves the sequential action of SPS and SPP, yielding free sucrose and inorganic phosphate. Cyanobacterial SPSs preferentially use ADP-glucose or UDP-glucose, as substrates. The disaccharide degradation can be carried out by the activities of the three different enzymes: (i) SuS that catalyzes a readily reversible reaction but that *in vivo* acts in the cleavage of sucrose, supplying ADP-glucose, a precursor for glycogen synthesis; however *in vitro*, SuS can also accept other sugar nucleotides (*i.e.*, UDP) as substrate; (ii) A/N-Inv that irreversible hydrolyzes sucrose into glucose and fructose; and (iii) AMS that is able to catalyze not only sucrose hydrolysis to hexoses, but also to transfer the glucose moiety to a soluble maltooligosaccharide or to an insoluble α 1,4-glucan.

The identification and functional characterization of sucrose-synthesis related enzymes was first described in *Anabaena* sp. PCC 7119, a filamentous heterocyst-forming strain [15]. Further studies were carried out in other model cyanobacteria, including *Synechocystis* sp. PCC 6803 (a freshwater unicellular strain) [16,17], *Anabaena* sp. PCC 7120 [18,19], *Synechococcus* sp. PCC 7002 (unicellular marine strain) [10], *Microcystis aeruginosa* PCC 7806 (a bloom-forming strain) [11], and *Synechococcus elongatus* PCC 7942 [12]. Basically, for sucrose-biosynthesis, it was shown a similar route to that of plants involving the sequential action of sucrose-phosphate synthase (SPS, U/ADP-glucose: d-fructose-6-phosphate 2-α-d-glucosyltransferase, EC 2.4.1.14) and sucrose-phosphate phosphatase (SPP, sucrose-6^F^-phosphate-phosphohydrolase, EC 3.1.3.24), yielding free sucrose and Pi (Figure 1). Cyanobacterial SPSs display important biochemical differences in comparison with the orthologous plant proteins. Thus, SPSs are not specific for UDP–glucose and most SPSs can accept ADP–glucose and, to a minor extent, other sugar nucleotides as substrates [15,16,19]. The hydrolysis of the intermediate by SPP leads to an essentially irreversible pathway providing an efficient production of sucrose even at low substrate concentrations [9,13].

On the other hand, sucrose utilization depends on the activity of three different enzymes: (i) sucrose synthase (SuS, A/UDP-glucose: d-fructose 2-α-d-glucosyltransferase, EC 2.4.1.13) that catalyzes a ready reversible reaction, but that, *in vivo*, is primarily involved in sucrose cleavage to supply sugar-nucleotide molecules and fructose [14,20,21]; (ii) alkaline/neutral invertase (A/N-Inv, an α-glycosidase, no EC number assigned yet), grouped in the glycoside family 100, which irreversibly hydrolyze the disaccharide into glucose and fructose [22]; and (iii) amylosucrase (AMS, EC 2.4.1.4), a member of family 13 of the glycoside hydrolases, described in bacteria, and recently reported in *Synechococcus* sp. PCC 7002 [23]. AMS is able to hydrolyze sucrose to glucose and fructose, and/or to transfer the glucose moiety to a maltooligosaccharide or α 1,4-glucan (amylose-like polymers).

**Figure 2:**
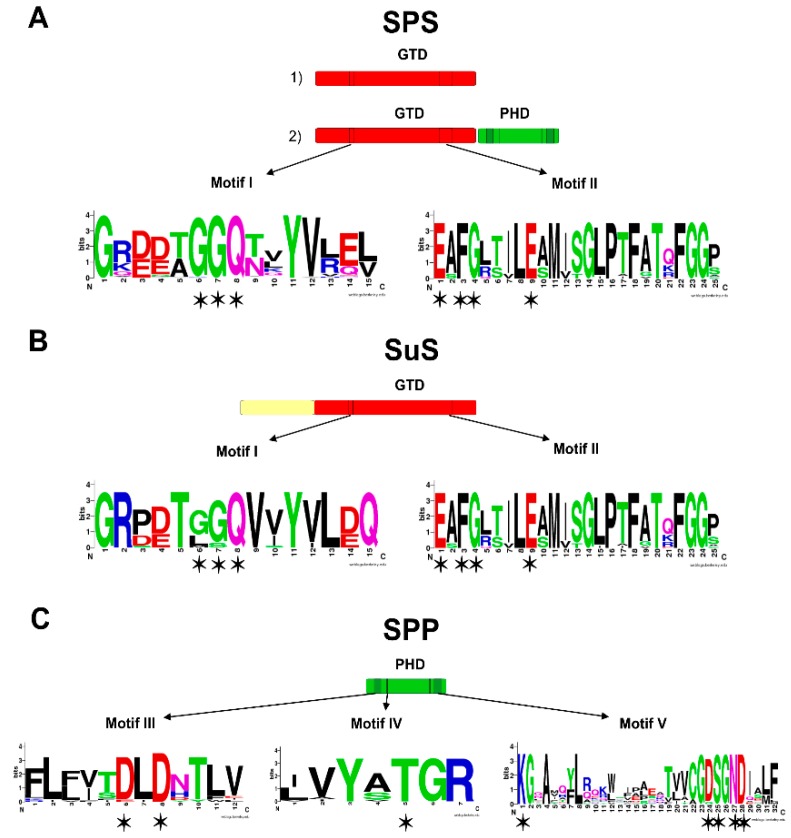
Domainal arrangements of sucrose-synthesis related proteins. SPS, SPP and SuS (sucrose-synthesis related proteins) are modular proteins based on a glycosyltransferase domain (GTD, red box) and a phosphohydrolase domain (PHD, green box) [9,19]. (**A**) Two domain arrangements have been described for cyanobacterial SPSs: (1) the minimal SPS unit (GTD), or unidomainal SPS; and (2) the two-domain SPS prototype (GTD-PHD) or bidomainal SPS; (**B**) SuS presents a GTD, with a characteristic N-terminal extension (yellow box). The resolution of the crystallographic structure of *Halothermothrix orenii* SPS (2r66A and 2r68A) [24], and of *Arabidopsis thaliana* SuS1 (3s28A) [25] allowed the identification of the residues involved in the sugar and in the NDP-glucose binding sites, within motif I and II, respectively (denoted with asterisks); (**C)** SPPs exhibit only a PHD. Motives III to V are characteristic of proteins grouped in the phosphohrydrolase superfamily and related to SPP activity. The crystallization *Synechocystis* sp. PCC 6803 SPP (1s2oA) led to the identification of the residues involved in the catalytic activity [26]. The critical residues were found within PHD motives (denoted with asterisks). Logos were constructed using the above mentioned conserved motives (WebLogo server [27]).

The functional characterization of the genes related to sucrose biosynthesis in unicellular and filamentous heterocyst-forming cyanobacteria have contributed to new insights into their structure, disclosing that SPS, SPP and SuS have a modular architecture [9]. The analysis of the two SPSs of *Anabaena* sp. PCC 7120 (SPS-A and SPS-B) uncovered an approximately 400 amino-acid region shared by all SPSs, allowing to define a functional glucosyltransferase domain (GTD) [19] (Figure 2). Similarly, the *Anabaena* SPP characterization [19] defined a phosphohydrolase domain (PHD) sharing conserved residues with other phosphohydrolases (Figure 2). SPSs support modularity since two different SPS domainal arrangements could be identified: the minimal SPS unit (GTD), coincidental with *Anabaena* SPSs, and the bidomainal SPS prototype (GTD-PHD), present in *Synechocystis* SPS, where the PHD is non-functional [19]. Additionally, the existence of bidomainal/bifunctional SPSs (exhibiting SPS and SPP activity) was demonstrated [12]. On the other hand, the analysis of SuS sequences also revealed that these proteins featured a GTD with a distinctive C-terminal extension [9].

### 2.2. The Ancestral Origin of Sucrose Biosynthesis

**Figure 3:**
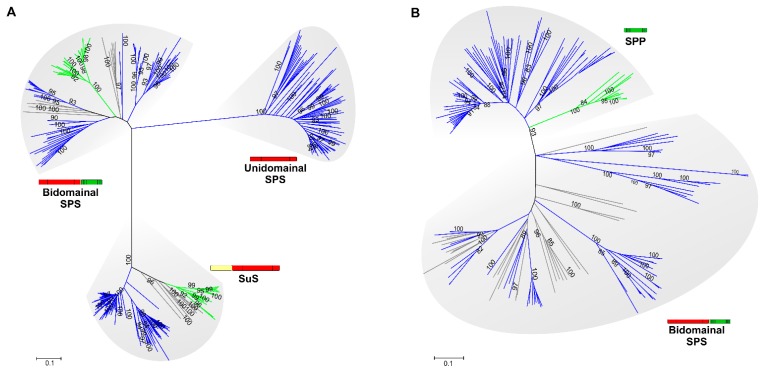
Phylogenetic analysis of SPS, SPP and SuS proteins based on GTD and PHD sequences. Homologs were retrieved from public databases (JGI-DOE, http://www.jgi.doe.gov) by BLASTp searches using as query SPS and SuS of *Anabaena* sp PCC 7120, and SPS and SPP of *Synechocystis* sp. PCC 6803. Unrooted dendrograms were obtained using the maximum parsimony (1000 replicates). After sequence alignments, GTD (A) or PHD (B) regions described by Cumino *et al.* [19] were identified with ClustalW [30]. Trees were generated with the MEGA5 software [31]. Major groups are identified to give clues about their function, species, or taxonomic information: (**A**) GTDs corresponding to bidomainal and unidomainal SPSs and SuS; (**B**) PHDs, corresponding to SPP and to bidomainal SPSs. Cyanobacteria, blue lines; plants, green lines; bacteria, grey lines. Bootstrap results are not shown when values were lower than 90%.

Earlier phylogenetic analyses based on GTD and PHD sequences revealed that sucrose biosynthesis proteins might have arisen from primordial functional domains shuffled during evolution [9], which was corroborated using sequences from 191 genomes available in May 2014 (Figure 3). The ancestral origin of sucrose metabolism postulated by Salerno and Curatti [9] was strongly supported by a recent study using ancestral sequence reconstruction coupled with phylogenetic analysis of sucrose synthesis genes [28]. In this report, it was hypothesized that sucrose synthesis in algae (chlorophytes and streptophytes) and land plants was likely inherited from cyanobacteria, and the chloroplast ancestor likely had the ability to synthesize sucrose [28,29]. However, sucrose metabolism genes were transferred to the nucleus, giving rise to a novel pathway in the plant lineage [9].

**Table 1:** Occurrence of homologous sequences to SPS, SPP, SuS and A/N-Inv encoding genes present in cyanobacterial genomes. BLAST searches were carried out using as query *Anabaena* sp. PCC 7120 SPS ^(1)^, *Synechocystis* sp. PCC 6803 SPS ^(2)^, *Anabaena* sp. PCC 7120 SPP ^(3)^, SuS ^(4)^, A/N-Inv ^(5)^, and *Synechococcus* sp. PCC 7002 AMS ^(6)^.

Order	Strain	Habitat	Place of origin	SPS (GTD) ^1^	SPS (GTD-PHD) ^2^	SPP ^3^	SuS ^4^	A/N-Inv ^5^	AMS ^6^
**Chroococcales**	*Acaryochloris marina* MBIC11017	Marine	Pacific Ocean	+	-	+	+	+	-
	*Acaryochloris* sp. CCMEE 5410	Aquatic	USA	+	-	+	+	+	-
	*Chamaesiphon minutus* PCC 6605	Aquatic	USA	2+	-	+	-	-	-
	*Crocosphaera watsonii* WH 0003	Marine	Pacific Ocean	-	-	-	-	-	-
	*Crocosphaera watsonii* WH 0003	Marine	Pacific Ocean	-	-	-	-	-	-
	*Crocosphaera watsonii* WH 0401	Marine	Atlantic Ocean	-	-	-	-	-	-
	*Crocosphaera watsonii* WH 8501	Marine	Atlantic Ocean	-	-	-	-	-	-
	*Crocosphaera watsonii* WH 8501	Marine	Atlantic Ocean	-	-	-	-	-	-
	*Cyanobacterium aponinum* PCC 10605	Freshwater	Italy	-	-	-	-	-	-
	*Cyanobacterium* sp. UCYN-A	Marine	Pacific Ocean	-	-	-	-	-	-
	*Cyanobacterium stanieri* PCC 7202	Freshwater	Chad	-	-	-	-	+	-
	*Cyanobium gracile* PCC 6307	Aquatic	USA	+	+	2+	-	-	+
	*Cyanobium* sp. PCC 7001	Marine	USA	+	+	+	-	+	-
	*Cyanothece* sp. BH63E, ATCC 51472	Aquatic	USA	-	-	+	-	-	-
	*Cyanothece* sp. BH68, ATCC 51142	Marine	USA	-	-	+	-	-	-
	*Cyanothece* sp. CCY 0110	Marine	Tanzania	-	-	+	-	-	-
	*Cyanothece* sp. PCC 7424	Freshwater	Senegal	2+	-	+	+	+	-
	*Cyanothece* sp. PCC 7425	Freshwater	Senegal	2+	-	+	2+	-	-
	*Cyanothece* sp. PCC 7822	Freshwater	India	+	-	+	-	+	-
	*Cyanothece* sp. PCC 8801	Freshwater	Taiwan	-	-	-	-	-	-
	*Cyanothece* sp. PCC 8802	Freshwater	Taiwan	-	-	-	-	-	-
	*Dactylococcopsis salina* PCC 8305	Aquatic	Israel	+	-	+	+	+	-
	*Geminocystis herdmanii* PCC 6308	Aquatic	USA	-	-	-	-	-	-
	*Gloeobacter kilaueensis* JS1	Freshwater	Hawaii	+	-	+	+	+	-
	*Gloeobacter violaceus* PCC 7421	Freshwater	Switzerland	+	-	+	+	-	-
	*Gloeocapsa* sp. PCC 73106	Freshwater	Switzerland	+	-	-	-	-	-
	*Gloeocapsa* sp. PCC 7428	Freshwater	Sri Lanka	-	-	+	2+	+	-
	*Halothece* sp. PCC 7418	Freshwater	Israel	2+	-	+	+	-	-
	*Microcystis aeruginosa* DIANCHI905/PCC7806	Freshwater	China/ Netherlands	+	-	+	+	-	-
	*Microcystis aeruginosa* NIES-843	Freshwater	Japan	-	-	-	-	-	-
	*Microcystis aeruginosa* PCC 7941	Freshwater	Canada	-	-	-	-	-	-
	*Microcystis aeruginosa* PCC 9432	Freshwater	Canada	-	-	-	-	-	-
	*Microcystis aeruginosa* PCC 9443	Freshwater	Africa	-	-	-	-	-	-
	*Microcystis aeruginosa* PCC 9701	Freshwater	France	-	-	-	-	-	-
	*Microcystis* aeruginosa PCC 9717	Freshwater	France	-	-	-	-	-	-
	*Microcystis aeruginosa* PCC 9806	Freshwater	USA	-	-	-	-	-	-
	*Microcystis aeruginosa* PCC 9807	Freshwater	South Africa	-	-	-	-	-	-
	*Microcystis aeruginosa* PCC 9808	Freshwater	Australia	-	-	-	-	-	-
	*Microcystis aeruginosa* PCC 9809	Freshwater	USA	-	-	-	-	-	-
	*Microcystis aeruginosa* SPC777	Freshwater	Brazil	-	-	-	-	-	-
	*Microcystis aeruginosa* TAIHU98	Freshwater	China	-	-	-	-	-	-
	*Microcystis* sp. T1-4	Freshwater	Thailand	-	-	-	-	-	-
	*Prochlorococcus marinus* AS9601	Marine	Arabian Sea	+	-	-	-	-	-
	*Prochlorococcus marinus* CCMP 1375	Marine	Atlantic Ocean	+	-	-	-	+	-
	*Prochlorococcus marinus* CCMP 1986	Marine	Mediterranean Sea	+	-	-	-	+	-
	*Prochlorococcus marinus* MIT 9211	Marine	Pacific Ocean	+	-	-	-	+	-
	*Prochlorococcus marinus* MIT 9215	Marine	Pacific Ocean	+	-	-	-	+	-
	*Prochlorococcus marinus* MIT 9301	Aquatic	Sargasso Sea	+	-	-	-	+	-
	*Prochlorococcus marinus* MIT 9303	Aquatic	Sargasso Sea	-	+	-	-	+	-
	*Prochlorococcus marinus* MIT 9312	Marine	Gulf Stream	+	-	-	-	+	-
	*Prochlorococcus marinus* MIT 9313	Marine	Gulf Stream	-	+	-	-	+	-
	*Prochlorococcus marinus* MIT 9515	Marine	Pacific Ocean	+	-	-	-	+	-
	*Prochlorococcus marinus* MIT9202	Marine	Pacific Ocean	+	-	-	-	+	-
	*Prochlorococcus marinus* NATL1A	Marine	Atlantic Ocean	-	+	-	-	+	-
	*Prochlorococcus marinus* NATL2A	Marine	Atlantic Ocean	-	+	-	-	+	-
	*Prochlorococcus* sp. CC9311	Marine	USA	-	+	-	-	+	-
	*Prochlorococcus* sp. CC9605	Marine	USA	-	+	-	-	+	-
	*Prochlorococcus* sp. CC9902	Marine	USA	-	+	-	-	+	-
	*Prochlorococcus* sp. W10	Marine	Pacific Ocean	-	-	-	-	+	-
	*Prochlorococcus* sp. W11	Marine	Pacific Ocean	-	-	-	-	+	-
	*Prochlorococcus* sp. W12	Marine	Pacific Ocean	-	-	-	-	-	-
	*Prochlorococcus* sp. W2	Marine	Pacific Ocean	+	-	-	-	-	-
	*Prochlorococcus* sp. W3	Marine	Pacific Ocean	-	-	-	-	-	-
	*Prochlorococcus* sp. W4	Marine	Pacific Ocean	-	-	-	-	+	-
	*Prochlorococcus* sp. W5	Marine	Pacific Ocean	-	-	-	-	-	-
	*Prochlorococcus* sp. W6	Marine	Pacific Ocean	-	-	-	-	-	-
	*Prochlorococcus* sp. W7	Marine	Pacific Ocean	+	-	-	-	+	-
	*Prochlorococcus* sp. W8	Marine	Pacific Ocean	+	-	-	-	+	-
	*Prochlorococcus* sp. W9	Marine	Pacific Ocean	-	-	-	-	+	-
	*Prochlorococcus* sp. WH 7803	Marine	Sargasso Sea	-	+	-	-	+	-
	*Prochlorococcu*s sp. WH8102	Marine	Atlantic Ocean	-	+	-	-	+	-
	*Prochlorothrix hollandica* PCC 9006	Freshwater	Netherlands	-	-	-	-	-	-
	*Rubidibacter lacunae* KORDI 51-2	Marine	Micronesia	+	-	+	+	-	-
	*Synechococcus elongatus* PCC 6301	Freshwater	USA	-	+	-	-	+	-
	*Synechococcus elongatus* PCC 7942	Freshwater	USA	-	+	-	-	+	-
	*Synechococcus* sp. BL107	Marine	Mediterranean Sea	-	+	-	-	+	-
	*Synechococcus* sp. CB0101	Marine	USA	-	+	+	-	+	-
	*Synechococcus* sp. CB0205	Marine	USA	-	+	+	-	+	-
	*Synechococcus* sp. CC9616	Marine	Pacific Ocean	-	-	-	-	+	-
	*Synechococcus* sp. JA-2-3B'a(2-13)	Freshwater	USA	-	-	+	-	+	-
	*Synechococcus* sp. JA-3-3Ab	Freshwater	USA	-	-	+	-	+	-
	*Synechococcus* sp. KORDI-100	Marine	South Korea	+	-	-	-	+	--
	*Synechococcus* sp. KORDI-49	Marine	South Korea	+	-	-	-	+	-
	*Synechococcus* sp. KORDI-52	Marine	South Korea	-	+	-	-	+	-
	*Synechococcus* sp. PCC 6312	Marine	USA	-	+	-	-	+	-
	*Synechococcus* sp. PCC 7002	Marine	Atlantic Ocean	-	+	-	-	-	+
	*Synechococcus* sp. PCC 7003	Marine	USA	-	+	+	-	-	+
	*Synechococcus* sp. PCC 7117	Marine	Asustralia	-	+	+	-	-	+
	*Synechococcus* sp. PCC 73109	Marine	USA	-	+	+	-	-	+
	*Synechococcus* sp. PCC 7335	Marine	Mexico	-	-	-	-	-	-
	*Synechococcus* sp. PCC 7336	Marine	USA	-	-	-	-	-	-
	*Synechococcus* sp. PCC 7502	Freshwater	Switzerland	+	-	+	-	+	-
	*Synechococcus* sp. PCC 8807	Freshwater	Gabon	-	+	-	-	-	+
	*Synechococcus* sp. RCC 307	Marine	Mediterranean Sea	+	-	+	-	+	-
	*Synechococcus* sp. RS9916	Marine	Israel	+	+	-	-	+	-
	*Synechococcus* sp. RS9917	Marine	Israel	-	+	-	-	+	-
	*Synechococcus* sp. WH 8016	Marine	USA	-	+	-	-	+	-
	*Synechococcus* sp. WH 8109	Marine	Sargasso Sea	-	+	-	-	+	-
	*Synechococcus* sp. WH5701	Marine	USA	+	-	+	-	+	+
	*Synechococcus* sp. WH7805	Marine	Sargasso Sea	-	+	-	-	-	-
	*Synechocystis* sp. PCC 6308	Freshwater	USA	-	+	+	-	-	-
	*Synechocystis* sp. PCC 6803	Freshwater	USA	-	+	+	-	+	-
	*Synechocystis* sp. PCC 7509	Freshwater	Switzerland	-	-	+	+	+	-
	*Thermosynechococcus elongatus* BP-1	Freshwater	Japan	-	+	+	+	-	-
**Oscillatoriales**	*Arthrospira maxima* CS-328	Freshwater	Chad	-	-	-	+	-	-
	*Arthrospira platensis* C1	Freshwater	Chad	-	-	-	+	-	-
	*Arthrospira platensis* NIES-39	Freshwater	Chad	-	-	-	+	-	-
	*Arthrospira platensis* Paraca	Freshwater	Peru	-	-	-	+	-	-
	*Arthrospira* sp. PCC 8005	Freshwater	India, Kenya, Mexico or Peru	-	-	-	2+	-	-
	*Crinalium epipsammum* PCC 9333	Unknown	Unknown	2+	-	+	+	+	-
	*Cyanobacterium* sp. ESFC-1	Freshwater	USA	2+	-	+	+	-	-
	*Geitlerinema* sp. PCC 7105	Freshwater	USA	2+	-	+	+	-	-
	*Geitlerinema* sp. PCC 7407	Freshwater	Unknown	+	-	2+	+	+	-
	*Leptolyngbya boryana* PCC 6306	Freshwater	USA	2+	-	+	2+	2+	-
	*Leptolyngbya* sp. 2LT21S03	Desert soil	Israel	-	-	+	-	-	-
	*Leptolyngbya* sp. PCC 6406	Freshwater	USA	-	-	-	-	-	-
	*Leptolyngbya* sp. PCC 7375	Freshwater	USA	-	-	-	-	-	-
	*Leptolyngbya* sp. PCC 7376	Freshwater	USA	-	-	-	-	-	-
	*Lyngbya majuscula* 3L	Marine	Netherlands Antilles	+	-	+	2+	+	-
	*Lyngbya* sp. CCY 8106	Freshwater	Germany	-	-	-	-	-	-
	*Microcoleus chthonoplastes* PCC 7420	Marine	USA	+	-	+	+	+	-
	*Microcoleus* sp. PCC 7113	Soil	USA	4+?	-	+	2+	2+	-
	*Microcoleus vaginatus* FGP-2	Desert soil	USA	+	-	+	+	-	-
	*Microcoleus vaginatus* PCC 9802	Soil crusts	USA	+	-	+	+	-	-
	*Nodosilinea nodulosa* PCC 7104	Soil	USA	-	-	-	-	-	-
	*Oscillatoria acuminata* PCC 6304	Soil	USA	2+	+	+	+	2+	-
	*Oscillatoria formosa* PCC 6407	Freshwater	USA	-	-	-	-	-	-
	*Oscillatoria nigro-viridis* PCC 7112	Soil	USA	+	-	+	+	-	-
	*Oscillatoria* sp. PCC 10802	Freshwater	Unknown	-	-	-	-	-	-
	*Oscillatoria* sp. PCC 6506	Freshwater	Unknown	-	-	-	-	-	-
	*Oscillatoriales* sp. JSC-1	Freshwater	USA	-	-	-	-	-	-
	*Oscillatoriales* sp. JSC-12	Freshwater	USA	2+	-	+	+	2+	-
	*Planktothrix agardhii* NIVA-CYA 126/8	Freshwater	Finland	-	-	-	-	-	-
	*Planktothrix agardhii* NIVA-CYA 34	Freshwater	Norway	-	-	-	-	-	-
	*Planktothrix agardhii* NIVA-CYA 56/3	Freshwater	Norway	-	-	-	-	-	-
	*Planktothrix* NIVA-CYA405	Freshwater	Norway	-	-	-	-	-	-
	*Planktothrix* NIVA-CYA406	Freshwater	Norway	-	-	-	-	-	-
	*Planktothrix prolifica* NIVA-CYA 540 (Draft1)	Freshwater	Norway	-	-	-	-	-	-
	*Planktothrix rubescens* NIVA-CYA 98	Freshwater	Norway	-	-	-	-	-	-
	*Planktothrix* sp. 585	Freshwater	Germany	-	-	-	-	-	-
	*Planktothrix* sp. NIVA CYA 15	Freshwater	Norway	-	-	-	-	-	-
	*Planktothrix* sp. NIVA-CYA 407	Freshwater	Norway	-	-	-	-	-	-
	*Planktothrix* sp. st147	Freshwater	Germany	-	-	-	-	-	-
	*Pseudanabaena* sp. PCC 6802	Freshwater	USA	+	-	+	+	+	-
	*Pseudanabaena* sp. PCC 7367	Marine	USA	-	-	+	+	-	-
	*Pseudanabaena* sp. PCC 7429	Freshwater	Switzerland	-	-	+	-	-	-
	*Spirulina major* PCC 6313	Brackish water	USA	-	-	-	-	-	-
	*Spirulina subsalsa* PCC 9445	Freshwater	Italy	-	-	-	-	-	-
	*Trichodesmium erythraeum* IMS101	Marine	USA	+	-	-	-	-	-
**Pleurocapsales**	*Chroococcidiopsis* sp. PCC 6712	Freshwater	USA	-	-	+	-	-	-
	*Chroococcidiopsis thermalis* PCC 7203	Soil	Germany	2+	-	+	+	2+	-
	*Pleurocapsa* sp. PCC 7319	Freshwater	Mexico	+	-	+	-	-	-
	*Pleurocapsa* sp. PCC 7327	Freshwater	USA	+	-	+	2+	2+	-
	*Stanieria cyanosphaera* PCC 7437	Freshwater	Cuba	2+	-	-	+	2+	-
	*Xenococcus* sp. PCC 7305	Marine	USA	-	-	+	-	-	-
**Nostocales**	*Anabaena circinalis* AWQC131C	Freshwater	Australia	+	-	+	-	-	-
	*Anabaena cylindrica* PCC 7122	Freshwater	United Kingdom	+	+	+	2+	+	-
	*Anabaena* sp. 90	Freshwater	Finland	+	+	2+	+	+	-
	*Anabaena* sp. PCC 7108	Freshwater	USA	+	+	+	+	+	-
	*Anabaena variabilis* ATCC 29413	Freshwater	USA	2+	-	+	2+	+	-
	*Calothrix desertica* PCC 7102	Sand	Chile	+	+	+	2+	4+	+
	*Calothrix* sp. PCC 6303	Freshwater	USA	+	+	+	+	+	-
	*Calothrix* sp. PCC 7103	Freshwater	USA	+	+	+	2+	4+	+
	*Calothrix* sp. PCC 7507	Freshwater	Switzerland	2+	-	+	2+	+	-
	*Cylindrospermopsis raciborskii* CS-505	Freshwater	Australia	+	-	+	-	+	-
	*Cylindrospermopsis raciborskii* CS-506	Freshwater	Australia	+	-	+	-	+	-
	*Cylindrospermopsis raciborskii* CS-509	Freshwater	Australia	+	-	+	-	+	-
	*Cylindrospermum stagnale* PCC 7417	Soil	Sweden	+	+	+	2+	+	-
	*Fremyella diplosiphon* UTEX 481	Freshwater	USA	-	+	2+	3+	2+	-
	*Microchaete* sp. PCC 7126	Freshwater	USA	2+		+	2+	+	-
	*Nodularia spumigena* CCY9414	Brackish water	Denmark	+	+	+	2+	2+	-
	*Nostoc azollae* 0708	Symbiont	Unkown	2+	-	+	+	+	-
	*Nostoc punctiforme* PCC 73102	Freshwater	Australia	2+	-	2+	2+	2+	-
	*Nostoc* sp. PCC 7107	Freshwater	USA		+	2+	2+	2+	-
	*Nostoc* sp. PCC 7120	Freshwater	USA	2+	-	+	2+	2+	-
	*Nostoc* sp. PCC 7524	Freshwater	Sri Lanka	+	+	+	2+	2+	-
	*Raphidiopsis brookii* D9	Freshwater	Brazil	-	-	-	-	-	-
	*Rivularia* sp. PCC 7116	Freshwater	USA	+	-	+	2+	+	-
	*Scytonema hofmanni* PCC 7110	Freshwater	Bermuda	2+	-	+	2+	2+	-
	*Scytonema hofmanni* UTEX 2349	Freshwater	USA	+	-	+	2+	+	-
**Stigonematales**	*Chlorogloeopsis fritschii* PCC 6912	Freshwater	India	+	-	+	2+	2+	-
	*Chlorogloeopsis fritschii* PCC 7702	Soil	India	+	-	+	2+		-
	*Chlorogloeopsis* sp. PCC 9212	Freshwater	Spain	+	-	+	2+	2+	-
	*Fischerella muscicola* PCC 7414	Freshwater	New Zealand	+	-	2+	3+	2+	-
	*Fischerella muscicola* SAG 1427-1	Freshwater	India	-	-	-	2+	+	-
	*Fischerella* sp. JSC-11	Freshwater	USA	+	-	+	2+	+	-
	*Fischerella* sp. PCC 9339	Freshwater	Unknown	+	-	+	-	2+	-
	*Fischerella* sp. PCC 9431	Freshwater	Unknown	2+	-	2+	+	+	-
	*Fischerella* sp. PCC 9605	Freshwater	Israel	+	-	2+	3+	2+	-
	*Fischerella thermalis* PCC 7521	Freshwater	USA	+	-	+	3+	+	-
	*Mastigocladopsis repens* PCC 10914	Soil	Spain	2+	-	+	2+	+	-
	*Mastigocoleus testarum* BC008	Marine	Puerto Rico	+	-	+	+	2+	-

Searches for homologs to sucrose-metabolism related genes revealed that they are not universal in modern strains (Table 1). While in most filamentous heterocyst-forming strains (Nostocales and Stigonematales), and in four out of six Pleurocapsales genomes, sucrose synthesis genes could be retrieved, they have lost in about 58% and 67% of the genomes of Chroococcales (unicellular strains) and Oscillatoriales (filamentous strains), respectively (Table 1). Blank [28] concluded that it is likely that multiple sucrose synthesis genes may have been present in the cyanobacterial ancestor, and that subsequent losses in many clades, gene duplication events in other groups (notably in Nostocales), and their regain by lateral gene transfer, might have occurred.

Regarding sucrose breakdown, homologs to SuS and A/N-Inv encoding genes are present in about 31% and 52%, respectively, of the genomes analyzed. In Chroococcales, there is a clear predominance of A/N-Inv for sucrose degradation with some particular exceptions (e.g., *Microcystis* strains). In contrast, homologs to SuS encoding genes could be retrieved mostly from genomes of heterocyst-forming cyanobacteria, and from a very few genomes of unicellular strains. Such is the case of the Chroococcales strains: *Gloebacter violaceus* PCC 7421, *Microcystis aeruginosa* PCC 7806, and *Thermosynechococcus elongatus* BP-1, where SuS encoding genes were functionally characterized [11]. The ancient origin of SuS in the cyanobacteria [9,28] is likely followed by gene duplications in Nostocales and several secondary gains in other strains. Thus, the occurrence of SuS in *G. violaceus*, which is the most deeply rooted cyanobacterium, seems to be a more recent lateral gene transfer event [11,28]. The fixation and long term persistence suggest that SuS might confer a selective advantage [32], such as the contribution to the ADP-glucose pool, which will be discussed in Section 3.2. In addition, the increase in SuS transcripts in *M. aeruginosa* and *G. violaceus* cells under hypoxic conditions led to speculate that this enzyme could be involved in the response to low oxygen conditions in some strains, which was also found in Arabidopsis [11,33].

It should be underlined that several *Synechococcus* and *Prochloroccocus* genomes only have bidomainal SPS (GTD-PHD) gene sequences, and lack an independent SPP sequence. As mentioned above, bidomainal SPSs with conserved key catalytic residues in the PHD module (e.g., *S. elongatus* PCC 7942) [12], could exhibit both SPS and SPP activity.

Regarding gene locations in cyanobacterial genomes, sucrose synthesis-related genes are separately located on different regions of the chromosome. However, sucrose transcriptional units were found in a few strains, such as in *Synechococcus* sp. PCC 7002 [10,23] and *Microcystis aeruginosa* [34]. These “sucrose clusters” are transcriptional units that contain sequences coding for SPS and SPP, as well as for sucrose breakdown proteins (SuS or AMS).

## 3. Sucrose Roles in Cyanobacteria

### 3.1. Sucrose as a Compatible Solute

Salinity is a key abiotic factor in aquatic ecosystems. In response to changes in external salt concentration, cyanobacteria have developed different protective mechanisms to maintain internal osmotic potential and to cope with the rise of cell ion concentration [35]. The two basic physiological responses for salt acclimation include the extrusion of toxic inorganic anions and the accumulation of organic compounds of low molecular mass, so-called compatible solutes for not interfering with cell metabolism. These osmolytes reduce the internal osmotic cell potential and prevent the denaturation of macromolecules induced by low water or high ionic concentrations [4]. Also, these molecules were shown to protect the cell from other types of desiccation, as well as from cold and heat stress [35,36,37].

Among cyanobacteria, a correlation between the nature of the principal organic solute and the strain-specific salt resistant level has been established [38]. In general, freshwater strains with low halotolerance (up to 0.7 M·NaCl), accumulate disaccharides such as sucrose and/or trehalose, as their major compatible solute, while cyanobacteria of moderate salt tolerance (up to 1.7 M·NaCl) synthesize mainly glucosylglycerol and glucosylglycerate. In some of these strains, sucrose can also be produced as a minor osmolyte [35,39]. Finally, halophilic strains tolerate up to 3 M·NaCl, and accumulate glycine betaine and glutamate betaine as compatible solutes [4].

The origin of the different osmolytes has been investigated by ancestral sequence reconstruction and phylogenetic analysis of the genes underlying salinity preferences in cyanobacteria [28]. This study concludes that the emergence of sucrose synthesis is likely to be ancestral in cyanobacteria, and associated with growth in a low salinity environment. Other osmolytes (glucosylglycerol, glucosylglycerate and glycine betaine) have emerged afterwards. The conclusions drawn also support the hypothesis of the freshwater origin of cyanobacteria followed by subsequent independent divergences into the marine environment.

As an osmoprotectant compound, sucrose (a polyhydroxyl molecule) can directly interact with macromolecules to achieve their stabilization through a mechanism accounted by the water replacement hypothesis, *i.e.*, by replacing at least part of the shell water around macromolecules [40]. The relevance of sucrose accumulation in salt acclimation was first shown in *Nostoc muscorum*, *Synechococcus* sp. PCC 6301 and *Anabaena variabilis* [5,41,42]. To date, the presence of sucrose as the main compatible osmolyte has been reported in many other freshwater strains [39] as well as in marine picocyanobacteria of the genera *Prochlorococcus* and *Synechococcus* [39].

Increase in sucrose accumulation in response to salt is due to higher SPS expression, as shown in filamentous nitrogen-fixing and unicellular strains [11,43,44,45]. For example, in *Anabaena* sp. PCC 7119 and 7120 cells, a short-term NaCl treatment resulted in a three-fold increase of SPS activity, which paralleled the rise of its polypeptide and transcript level [46,45]. Similar results were obtained in *Synechocystis* sp. PCC 6803 and *M. aeruginosa* PCC 7806 [34,47]. Interestingly, in the marine strain *Synechococcus* sp. PCC 7002, the addition of 684 mM·NaCl increased the expression of SPS and SPP encoding genes organized in a transcriptional unit (sucrose cluster), whose promoter region contains a consensus motif characteristic of osmotic and salt-activated genes [10]. Recently, in *Anabaena* sp. PCC 7120, it has been shown that OrrA, a NarL-type response regulator [48], is necessary to induce the genes involved in sucrose synthesis in response to salt stress [49].

Remarkably, after salt addition, not only the expression of sucrose synthesis proteins but also that of sucrose degradation enzymes increased in *Anabaena* sp. filaments, and in *M. aeruginosa* sp. PCC 7806 and *G. violaceus* PCC 7421 cells. Such effect on both, sucrose synthesis and breakdown, could be ascribed to a “sucrose cycling”, as reported by Cumino *et al.* [11,34,45].

It is worth noticing that, in addition to sucrose, novel compatible solutes (soluble polymers named sucroglucans) have been recently identified in *Anabaena* sp. PCC 7119, PCC 7120, and *A. variabilis.* These oligosaccharides reversibly accumulate in the first hours after exposure to NaCl [46], and constitute a series of non-reducing sucrose derivatives, where glucose is linked, through its hemiacetalic hydroxyl, to the 2 position of the glucose moiety of sucrose. The members of the sucroglucan series should be included in the repertoire of osmolytes synthesized in response to salt in low halotolerance filamentous heterocyst-forming strains [50].

However, what is more surprising is that sucrose appears to have a more intricate function than to being an osmolyte according to experiments reported for *Synechocystis* sp. PCC 6803. In this unicellular moderately halotolerant strain, the dominating osmoprotective compound is glucosylglycerol and sucrose was considered as a secondary osmolyte, either in salt-adapted or salt-shocked cells [35,44,51]. However, by examining the time-course of sucrose production in cells subject to a salt stress, a transient accumulation of the disaccharide was shown at the onset of treatment [47]. Intracellular sucrose concentration increased immediately after the NaCl shock, reaching its peak between 4 h and 6 h after the onset, and decreasing sharply in the following 20 h up to approximately the initial level. In line with that result, the steady-state amount of SPS gene transcripts increased very quickly after the salt shock, reaching a maximum after 30 min and returning to the initial levels during the following hour. It should be stressed that sucrose production kinetics contrasts with the typical accumulation pattern of an osmolyte like glucosylglycerol. Moreover, the analysis of a *Synechocystis* mutant strain impaired in sucrose synthesis showed that sucrose presence was essential in stationary phase cells to overcome a subsequent salt stress, but that it was dispensable for growth under standard conditions. In few words, Desplats *et al.* [47] accounted for the first demonstration of sucrose playing a role other than being a compatible osmolyte for salt tolerance and suggested that the disaccharide was likely acting as a signal molecule.

### 3.2. Sucrose and Nitrogen Fixation

Many cyanobacteria are able to simultaneous and independently produce photosynthetic molecular oxygen and fix atmospheric nitrogen (N_2_). Certainly, nitrogen fixation into ammonia is an energetically expensive process always linked to carbohydrate metabolism [52]. Because nitrogenase, the enzymatic system for N_2_ fixation, is inactivated upon exposure to oxygen, cyanobacteria have evolved different adaptations that include either temporal or spatial separation of the two processes [53]. Particularly, under aerobiosis and combined-nitrogen withdrawn, cyanobacteria clustered in phylogenetically-coherent groups of filamentous strains (Nostocales and Stigonematales) are able to differentiate a photosynthetic (vegetative) cell into a specialized cell called heterocyst, through a variety of structural, biochemical, and genetic changes, allowing the nitrogenase to be active [54,55,56]. Heterocysts, distributed in a semiregular pattern along the filaments [57], contain the oxygen-sensitive enzyme complex nitrogenase and lack photosystem II activity and ribulose-1,5-diphosphatecarboxylase (RuBisCo), a key enzyme in CO_2_ fixation during photosynthesis. Consequently, heterocysts are limited to a heterotrophic metabolism and depend on vegetative cells for the generation of carbon skeletons and reducing power [58,59,60].

Despite the fact that several attempts have been made to elucidate the carrier molecule/s responsible for transporting the reduced carbon from vegetative cells to heterocysts, it remains to be precisely identified. Several carbohydrates, including fructose, erythrose and sucrose, have been suggested as possible carriers [8,59,61]. Sucrose, as a transport molecule, was proposed based on SuS and invertase activities measured in *Anabaena variabilis* cell extracts [8]. However, this transport was not demonstrated and the function of SuS was shown to be sucrose cleavage [20,62].

The critical role of sucrose in carbon flux modulation in the nitrogen-fixing filaments of *Anabaena* sp. was undoubtedly proven in subsequent studies. Curatti *et al.* [63] showed that diazotrophic growth was impaired in an *Anabaena* mutant strain overexpressing the SuS encoding gene (*susA*), in which the disaccharide was not detectable. These results support the SuS involvement in the control of carbon flux in vegetative cells through the cleavage of sucrose. Indeed, it was a clear demonstration that SuS preferentially catalyzes the cleavage of the disaccharide *in vivo*. Afterwards, it was shown in heterocysts that the expression of SuS and RuBisCo is similarly down-regulated by a nitrogen source-dependent developmental program [62].

The analysis of complete sequenced genomes indicates that most heterocyst-forming strains have at least two homologs to SPS genes and two homologs to A/N-Inv genes (Table 1). The existence of SPS-A and SPS-B, and Inv-A and Inv-B in *Anabaena* sp. PCC 7120 [15,19,64] has raised the question of whether each isoenzyme could play distinct roles in the nitrogen-fixing filaments. Analyses of cellular localizations of the isoforms, phenotypes of insertional mutants, and studies on the transcriptional regulation of the sucrose protein encoding genes, have provided conclusive evidence supporting the role of the disaccharide as an intermediate in the reduced carbon flux along the N_2_-fixing filaments. Furthermore, it was shown that while both SPSs and both A/N-Inv contribute to sucrose metabolism in vegetative cells, only SPS-B and Inv-B are active in heterocysts [20,65,66]. These enzymes are likely to be part of a sucrose cycling inside the heterocyst, playing essential functions in carbon–nitrogen balance. Cumino *et al.* [20] proposed that a sucrose cycling mechanism may be operating in the heterocysts, allowing cell metabolism to shift easily from sucrose production to degradation through A/N-Inv, hexokinase, hexose-P mutase, hexose-P isomerase, ADP-glucose pyrophosphorylase (AGPase, a key enzyme involved in glycogen synthesis), SPS-B, and sucrose-phosphate phosphatase activities. Even though the role of glycogen in nitrogen fixation has been studied for several decades [67,68,69,70,71,72], the interconnection between glycogen metabolism and sucrose in nitrogen-fixing filaments was demonstrated by the integration of results of expression and metabolic flux analyses of sucrose metabolism enzymes. This allowed proposing that a sucrose cycling is linked to glycogen metabolism and respiration [20]. According to sucrose metabolic network modeling in nitrogen-fixing *Anabaena* filaments, AGPase flux alone (calculated by metabolic simulation) was insufficient to supply the substrate (ADP-glucose) for glycogen and sucrose synthesis. Then, it was proposed that sucrose cleavage by SuS in the vegetative cells would contribute to the ADP-glucose pool [20]. Moreover, SuS was shown to be involved in the sucrose to insoluble polysaccharides conversion according to nutritional and environmental signals in *Anabaena* filamentous under diazotrophic growth [21].

The coordination at the transcriptional level of sucrose metabolism with nitrogen assimilation was supported by experimental evidence indicating that NtcA (a global nitrogen regulator in cyanobacteria, required for the expression of proteins subject to ammonium repression [73]) also regulates sucrose metabolism genes in *Anabaena* sp. PCC 7120. NtcA acts as a transcriptional activator of the encoding genes of SPS-B and Inv-B (both proteins located into heterocyst), and as an inhibitor of SuS encoding gene [65,66,74]. NtcA is required to maintain a high sucrose biosynthesis and a low rate of the disaccharide cleavage in the vegetative cells. Also, in the heterocyst, it regulates both sucrose synthesis by SPS-B and its hydrolysis by Inv-B. In mutants where the Inv-B encoding gene (*invB*) was knocked out, the filaments were unable to grow on diazotrophic conditions and the accumulation of sucrose and glycogen was altered [65]. These results demonstrate an essential role for Inv-B for diazotrophic growth and that Inv-B plays a key part in the coordination of sucrose and glycogen metabolism. It appears that NtcA integrates signals from carbon and nitrogen metabolism, and regulates gene expression accordingly to redirect metabolism as a function of the carbon/nitrogen status of the cell. Therefore, an expanded role as a global metabolism regulator was proposed for NtcA [74].

## 4. Final Remarks

**Figure 4:**
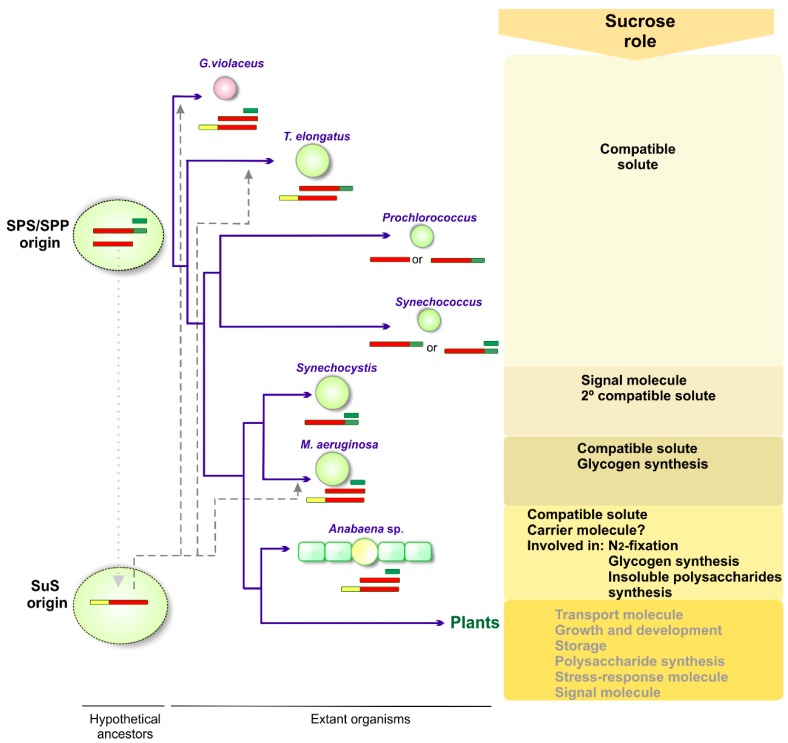
Schematic representation of sucrose roles along the hypothetical evolutionary pathway of cyanobacteria. The phylogenetic relationships among species are depicted according to *rDNA 16S* sequence analysis. Sucrose metabolism is likely to be originated in freshwater habitat and multiple sucrose synthesis genes might have been present in a cyanobacterial ancestor [27]. Sucrose synthesis is found in *G. violaceus* that has ancestral characteristics and diverged early within the radiation of cyanobacteria. A fusion of primordial GTD and PHD might have given rise to a hypothetical common-ancestral SPS (GTD-PHD) gene, which is found mostly in the marine *Prochlorococcus/Synechococcus* clade. Sucrose has been identified as a primary compatible solute in *Prochlorococcus*, and as secondary osmolyte in *Synechococcus* strains and in *Synechocystis* sp. PCC 6803 [36]. The involvement of sucrose in glycogen and polysaccharides production seems to be due to the emergence of SuS (dotted line), crucial in filamentous heterocyst-forming strains [20,21], as well as in strains (such as *G. violaceus*, *Thermosynechococcus* elongatus and *Microcystis* *aeruginosa* PCC 7806), where SuS are likely to be acquired by lateral gene transfer (dashed lines). In heterocystic strains, sucrose is a key molecule during nitrogen fixation and it was proposed as a carrier molecule to transport carbon along the filament. It is also involved in glycogen synthesis and in other polysaccharide accumulation. Plant sucrose metabolism has been acquired during the endosymbiotic origin of the chloroplast at the time of the cyanobacterial phylogenetic radiation.

In the last two decades, a comprehensive set of data has contributed to reveal that sucrose, aside from being a compatible solute in response to salinity, can play other crucial roles in the life of many cyanobacteria. Figure 4 summarizes the current state of knowledge of sucrose physiological functions in modern cyanobacteria and how they are related to cyanobacterial phylogeny and to the occurrence of sucrose synthesis proteins.

The emergence of sucrose as a compatible solute is likely to be ancestral and to have occurred in freshwater strains where cyanobacteria might have originated and the synthesis of the disaccharide was sufficient to withstand low salinity environments. The subsequent acquisition of the synthesis of new compatible solutes by unicellular strains conferred them moderate tolerance to salinity, and it is likely that sucrose was shifted into a secondary role as osmolyte. This would be the case of *Synechocystis* sp. PCC 6803, where glucosylglycerol is the main osmolyte. However, in this strain a new function for sucrose was made evident. Whether the low and transient accumulation of sucrose (typical of a signal molecule) in *Synechocystis* could be a more general feature in cyanobacteria should be further explored.

In addition to its role as a stress-response molecule, in filamentous nitrogen-fixing strains, sucrose metabolism is crucial for the heterocyst function, in glycogen accumulation, and in the flux of carbon between sucrose and polysaccharides.

Despite the fact that the capability of sucrose synthesis is likely to be ancestral and mostly universal in extant cyanobacteria, it seems not to be essential for the survival of many strains, as it was lost in many clades and mutants impaired in sucrose synthesis could be isolated. However, the acquisition of sucrose synthesis genes by lateral transfer could point to some adaptive advantage to occupy new ecological niches.

We wonder which might have been the selective advantage of sucrose that led to such an evolutionary choice in the cyanobacterial lineage.
